# *HNF4A*-related Fanconi syndrome in a Chinese patient: a case report and review of the literature

**DOI:** 10.1186/s13256-018-1740-x

**Published:** 2018-07-14

**Authors:** Jiaojiao Liu, Qian Shen, Guomin Li, Hong Xu

**Affiliations:** Department of Nephrology, Children’s Hospital of Fudan University, Shanghai, China

**Keywords:** Fanconi syndrome, *HNF4A*, Hyperinsulinemic hypoglycemia, Hearing loss, Gene analysis

## Abstract

**Background:**

The p.R63W mutation in hepatocyte nuclear factor-4 alpha (*HNF4A*) leads to a heterogeneous group of disorders with various clinical presentations. Recently, patients with congenital hyperinsulinism and Fanconi syndrome due to the p.R63W mutation in *HNF4A* have been described. Although other clinical variations such as liver dysfunction have been associated with *HNF4A* mutations, hearing impairment has not previously been associated. We report the case of a patient with Fanconi syndrome and hyperinsulinemic hypoglycemia caused by the mutation of *HNF4A* presenting with additional auditory phenotypes.

**Case presentation:**

We present a case report of a 10-year-old girl of Chinese Han ethnicity who presented with renal Fanconi syndrome, infantile hyperinsulinemic hypoglycemia, and transient cholestasis. In addition, she presented with bilateral severe hearing loss. Gene analysis showed a heterozygous p.R63W mutation in the *HNF4A* gene that is responsible for Fanconi syndrome and hyperinsulinemic hypoglycemia.

**Conclusions:**

This is the first case of *HNF4A* mutation associated with an auditory phenotype. It expands the clinical phenotypes and supports speculation in the literature that *HNF4A* may be a candidate gene for deafness. In conclusion, hearing loss may be found in children with *HNF4A*-related Fanconi syndrome, and auditory function should be assessed.

## Background

Renal Fanconi syndrome (FS) is a consequence of decreased solute and water reabsorption in the proximal tubule of the kidney. The common clinical manifestation is polydipsia and polyuria with phosphaturia, glycosuria, and aminoaciduria. In children, FS often occurs as part of a multisystem metabolic disease originating from heterogeneous genetic deficiencies. Recently, FS caused by mutations in the hepatocyte nuclear factor-4 alpha (*HNF4A*) gene was reported by Hamilton *et al.* [1], as a clinical syndrome accompanied by hyperinsulinemic hypoglycemia and early-onset diabetes.

Here, we report a case of FS caused by *HNF4A* mutation that presented with hearing loss. To the best of our knowledge, this is the first report of *HNF4A*-related FS associated with an additional auditory phenotype, adding to the scope of the disease.

## Case presentation

A 10-year-old girl was referred to the Children’s Hospital of Fudan University because of polydipsia and polyuria. She was born to non-consanguineous healthy parents of Chinese Han ethnicity and good socioeconomic status. She was the only child of the family, and there was no family history of FS. Newborn hearing screening failed. There was no history of birth defects. She was not receiving any medication and did not take alcohol or smoke tobacco. She was in Grade 4 of primary school and was not good at studying.

At 3 months of age, she presented with jaundice, hepatomegaly (3.5 cm below the costal margin), and splenomegaly (4 cm below the costal margin). She was admitted to our hospital. Laboratory findings revealed: elevation of direct bilirubin (DB), that is, total bilirubin (TB) 66.1 μmol/L (normal range, 0–6 μmol/L) and DB 61.4 μmol/L (normal range, 5.1–17.6 μmol/L); and almost normal transaminases, that is, alanine aminotransferase (ALT) 24 IU/L (normal range, 0–40 IU/L) and aspartate aminotransferase (AST) 46 IU/L (normal range, 0–40 IU/L)). Laboratory tests for hepatotropic viruses were negative. Magnetic resonance cholangiopancreatography excluded bile duct obstruction. After treatment with ursodiol, the jaundice resolved gradually. During the follow-up years, her liver functions were normal. Hypoglycemia was initially noticed during hospitalization, and fasting blood glucose ranged from 1.4 to 2.8 mmol/l. Prior to this, there was no record of a hypoglycemic episode. At the time of hypoglycemia (blood glucose 1.4 mmol/l), an inappropriate glycemic response to glucagon (increase of 4.3 mmol/l) was consistent with excess insulin action, confirming hyperinsulinism. Frequent feeding combined with intravenously administered glucose (6–7 mg/kg per minute) was required to maintain normoglycemia. She was discharged from the nursery with stable glucose levels (4.3–6.5 mmol/l) on the condition of frequent feeds. At 1 year of age, she experienced an episode of hypoglycemia, and the symptoms resolved after feeding. Subsequently, no symptoms of hypoglycemia appeared again.

Short stature was noticed (height and weight were below the third percentile) by routine physical examination at the age of 5. Laboratory investigations revealed proteinuria and glycosuria, mild acidosis, and hypophosphatemic rickets. Prior to this, she had recurrent urinary tract infections, suggesting an earlier onset of abnormal urine. Based on the clinical impression of FS, she was treated with calcitriol, phosphorus, and potassium citrate. Over the ensuing years, sodium citrate and other medications were used to maintain acid-base balance, but there was no improvement in her short stature, and her serum creatinine remained elevated by age 10.

**Fig. 1:**
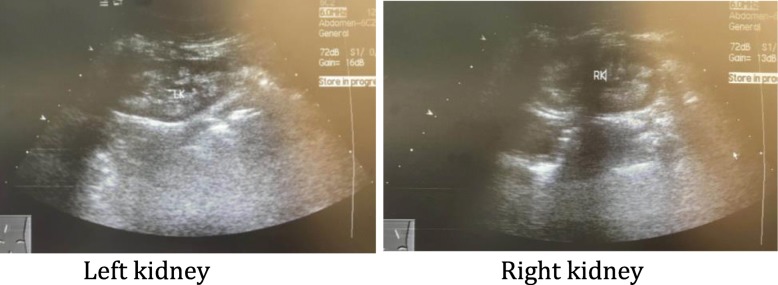
Renal ultrasonographic images show increased reflectivity of the renal pyramids, which demonstrate nephrocalcinosis

At 10 years of age, she was admitted to our clinic because of symptoms of FS and elevated serum creatinine: 75 μmol/L (normal range, 21–65 μmol/L). On this admission, her height was 126.5 cm (below the third percentile) and her weight was 28 kg (25th percentile). Laboratory investigations revealed: renal glycosuria in the absence of hyperglycemia; proteinuria with a urinary protein to creatinine ratio (pro/Cr) of 2.17 (normal range, 0–0.2); hypercalciuria with a urinary calcium to creatinine ratio (U-Ca/Cr) of 0.31 (normal range, < 0.21); and hypouricemia with 66 μmol/L (normal range, 90–420 μmol/l). Fasting glucose and post-prandial glucose were normal. No known cause for FS was identified. Glomerular filtration rate (GFR) was 55.7 ml/minute/1.73 m^2^ measured by ^99m^Tc-diethylenetriaminepentacetate (DTPA) renal dynamic imaging. Renal ultrasonography showed nephrocalcinosis (Fig. 1). A radiological examination showed complete recovery of rickets. Pure tone audiometry revealed a bilateral hearing loss of more than 50 dB. Because of multisystem involvement, she underwent whole exome sequencing and mutational analysis, revealing a heterozygous p.R63W mutation in the *HNF4A* gene. Both parents tested negative for the mutation. No genes associated with deafness phenotypes were found.

## Discussion

We reported the case of a patient carrying the *HNF4A* p.R63W mutation, who presented with FS, infantile hyperinsulinemic hypoglycemia, and transient cholestasis, similar to reported cases in the literature [1–6]. In addition, our patient had bilateral severe hearing loss.

The link between *HNF4A* p.R63W mutation and FS was first described by Hamilton *et al.* in 2014 [1]. The authors proposed that the *HNF4A* p.R63W mutation was a mutation specific for renal FS. Subsequently, more than 14 patients from 11 families have been described worldwide [1–6]. Most of these patients presented with hyperinsulinism, hypoglycemia, renal proximal tubular dysfunction, and liver involvement. Hyperinsulinism, hypoglycemia, and FS are frequently reported clinical phenotypes with this mutation. Almost all the patients carried the p.R63W mutation and had the pancreatic β-cell phenotype as well as the renal phenotype [1–6]. Phenotypic characteristics of our patient were similar to those reported in the literature except for hearing loss. A total of 14 patients with the p.R63W mutation have been described, and their clinical phenotypes are summarized in Table 1.

**Table 1:** Clinical phenotypes of patients with the *HNF4A* p.R63W mutation

Family	I	II	II	II	III	IV	V	VI	VII	VIII	IX	X	XI	XI
Patient No	1	2	3	4	5	6	7	8	9	10	11	12	13	14
Current age	N/A	30y	29y	5y	39y	17y	7y	N/A	N/A	10 m	Died	N/A	N/A	N/A
Sex	F	F	F	M	F	F	F	M	M	M	M	M	F	M
Macrosomia	–	+	+	+	–	+	–	+	+	–	–	+	–	–
Hypoglycemia (onset age)	+, Day1	+, Neonatal	+, Neonatal	+, Neonatal	+, Neonatal	+, Neonatal	+, Neonatal	+, Day1	+, Day1	+, Day2	+, Neonatal	+, Day1	+, Neonatal	+, Neonatal
Treatment and duration	Diazoxide, 4 years	Intravenous glucose, 1 week	Intravenous glucose, 3 days	Diazoxide, 2 weeks	Intravenous glucose	Diazoxide, 4 years	Diazoxide, 6 months	Diazoxide	Intravenous glucose, 17 days	Diazoxide, 10 months	Diazoxide, 7 months (died)	Diazoxide, 3 months	N/A	N/A
Diabetes (age)	–	–	–	–	+, (20 years)	+, (12 years)	–	–	–	–	–	–	–	–
Liver involvement (onset age)	+, 3 m	–	–	–	–	–	–	–	+, 7 m	+, Neonatal	–	+, 6 m	+, Neonatal	–
Presentation	Hepatomegaly and elevated transaminases	–	–	–	–	–	–	–	Hepatomegaly and elevated transaminases	Elevated conjugated bilirubin	–	Hepatomegaly and elevated transaminase	Jaundice and hepatomegaly	–
Liver biopsy	Abundant cytoplasmic glycogen	–	–	–	–	–	–	–	–	–	–	–	+, Normal	–
Fanconi syndrome (onset age)	+, 1y	+, 25y	+, 23y	+, Neonatal	+, 3y	+, 4y	+, 4y	+, 4 m	+, 8 m	+, Neonatal	+, Neonatal	+, 18 m	+, 3y	+, Neonatal
Growth retardation	N/A	+	+	+	+	+	+	N/A	+	N/A	N/A	+	+	N/A
Rickets	+	+	+	N/A	N/A	N/A	N/A	+	–	N/A	N/A	+	+	–
Nephrocalcinosis	N/A	+	+	+	+	+	+	–	–	–	–	N/A	+	N/A
eGFR	N/A	47	39	42	23	60	62	N/A	N/A	N/A	N/A	N/A	47	N/A
Ref.	Stanescu *et al*. [2]	Hamilton *et al*. [1]	Hamilton *et al*. [1]	Hamilton *et al*. [1]	Hamilton *et al*. [1]	Hamilton *et al*. [1]	Hamilton *et al*. [1]	Numakura *et al*. [3]	Numakura *et al*. [3]	Improda *et al*. [4]	Improda *et al*. [4]	Clemente *et al*. [5]	Walsh *et al*. [6]	Walsh *et al*. [6]

*eGFR* estimated glomerular filtration rate, *F* female, *m* month, *M* male, *N/A* not available, *y* year, *+* yes, *−* no

*HNF4A* mutations can cause hyperinsulinemic hypoglycemia and macrosomia in the neonatal period, as well as diabetes in adolescence or early adulthood [7, 8] caused by an impaired insulin secretory response to glucose in pancreatic β-cells [9]. Increased insulin secretion in neonates and decreased insulin secretion in later life might explain the hypoglycemia and diabetes at various stages of the disease, but the mechanism of the differential aspects of the *HNF4A* defect on β-cell function is unclear. In these reported case series, 100% (14/14) of the patients with *HNF4A* p.R63W mutations had infantile hyperinsulinemic hypoglycemia and 50% (7/14) were macrosomic (Table 1). The severity and duration of hyperinsulinemic hypoglycemia was variable, with some patients requiring diazoxide therapy for several years, while some required intravenously administered glucose for days. Most of the patients experienced hypoglycemia in the neonatal period, suggesting that it is essential to monitor glucose in neonates. Two of the 14 patients developed diabetes at 12 and 20 years of age [1]. To date, diabetes has not developed in our patient. Oral glucose tolerance testing is necessary for *HNF4A* mutation carriers during follow-up.

According to Hamilton *et al.* [1], FS is a mutation-specific phenotype associated with the p.R63W mutation, and the penetrance of FS in patients with the mutation is complete. The mechanism of the mutation-specific phenotype is unclear; it was hypothesized that the mutant *HNF4A* altered DNA binding and influenced the interaction with major regulatory genes; however, the details of target genes are not known [1, 9]. All 14 patients who were reported to be carrying this particular mutation presented with typical or atypical FS. The youngest onset age of *HNF4A*-related FS reported was the neonatal period, with 85% (12/14) diagnosed by age 4. The usual complications of FS are growth retardation and rickets in children. In these case series, 64% (9/14) had growth retardation or short stature, and 42% (6/14) had rickets. Supplementation of substances lost in the urine (primarily phosphate and bicarbonate) is the primary treatment and can induce rickets remission and catch-up growth. Short stature and rickets also occurred in our patient, and sodium citrate, potassium, phosphate, and calcitriol treatment led to remission of the rickets. The prognosis for this disease is not optimistic, with half of the patients progressing to stage II–IV chronic kidney disease (CKD). Renal function impairment was also observed in our patient, with a GFR of 55.7 ml/minute/1.73 m^2^. Further follow-up is necessary to assess the renal function and monitor for progression of CKD in those patients.

Liver phenotypic variability and incomplete penetrance are seen in patients with the R63W mutation. Approximately 35.7% (5/14) of the patients with this mutation presented with variable liver phenotypes, including hepatomegaly, jaundice, elevated transaminase, and increased glucose stores. Our patient presented with hepatomegaly and jaundice, similar to the case reported by Improda *et al.* [4]. Most of the liver dysfunction is transient. It is obvious that this mutation does not lead to severe liver disease.

Our patient presented with severe hearing loss. To date, 11 patients have been reported to be carrying the p.R63W mutation in the *HNF4A* gene, and in none of them was auditory status mentioned. The association of *HNF4A* defects with hearing loss has not been previously described. Stamatiou and Stankovic [10] identified a number of molecules including *HNF4A* that are likely to be key mediators of genetic hearing loss through network and pathway analyses. According to their report, *HNF4A* may be a new candidate gene for deafness. Our hypothesis is that mutant *HNF4A* may play an important role in hearing loss. On the one hand, *HNF4A* is a member of the steroid hormone receptor superfamily of ligand-dependent transcription factors that regulates hundreds of target genes. Based on the fact that the R63 residue is in the DNA-binding domain and directly contacts the DNA response element, we speculated that the heterozygous p.R63W mutation in our patient reduced DNA binding activity and altered expression of some target genes related to otic capsule development or associated hearing loss; on the other hand, according to Stamatiou and Stankovic [10], *HNF4A* appeared to be a central player in the network and was an attractive gene for hearing loss based on network analysis of human deafness. Further studies will be needed to clarify the association between *HNF4A* defects and auditory phenotypes.

Taken together, the specific mutation in *HNF4A* caused a group of disorders, including hypoglycemia, renal FS, and liver dysfunction, similar to Fanconi–Bickel syndrome, a rare type of glycogen storage disease caused by solute carrier family 2 member 2 (*SLC2A2*) defects with which FS is easily confused. The most noticeable difference between them is that the hypoglycemia of the former is due to hyperinsulinemia, whereas that of the latter is due to impaired hepatic release of glucose [11].

## Conclusions

To the best of our knowledge, this is the first case of an *HNF4A* mutation associated with auditory phenotype, expanding the range of clinical phenotypes and supporting speculation in the literature that *HNF4A* may be a candidate gene for deafness.

In conclusion, in a case of clinically diagnosed FS with a history of neonatal hyperinsulinemic hypoglycemia, the p.R63W mutation in *HNF4A* should be suspected. Considering multisystem involvement, close observation of liver function, auditory condition, and blood glucose levels is suggested for these patients, all of which may occur in various courses of the disease.
